# 

**Published:** 1844-10

**Authors:** 


### CONTENTS OF No. XXXVI

OF THE

### British and Foreign Medical Review.

### OCTOBER, 1844.

---

### Part First.—Analytical and Critical Reviews.

PAGE

Art. I.—1. Guide de Médecin Praticien, ou Résumé général de Pathologie Interne et de Thérapeutique appliquées. Par F. L. J. Valleix, Médecin du Bureau central des Hôpitaux de Paris, &c. Tome I . . . 285

Guide of the Practical Physician, or General Summary of Internal Pathology and Therapeutics, clinically applied. By F. L. J. Valleix, Physician to the Central Bureau of Hospitals of Paris. Vol. I.

2. The Practice of Medicine; a Treatise on Special Pathology and Therapeutics. By R. Dunglison, m.d., Professor of the Institutes of Medicine in Jefferson Medical College, Philadelphia. Second Edition . . . ib.

Art. II.—Diseases of the Lungs from Mechanical Causes, and Inquiries into the condition of the Artisans exposed to the Inhalation of Dust. By G. Calvert Holland, m.d., Physician Extraordinary to the Sheffield General Infirmary 302

Art. III.—Praxeos Medicæ Universæ Præcepta. Auctore Josepho Frank, Joannis Petri Filio, &c. Partis Tertiæ Volumen Secundum, Sectio Prima, continens Doctrinam de Morbis Tubi Intestinalis. Quam exposuit Fred. Augustus Benj. Puchelt, Magno Duci Badarum à Consiliis Aulæ intimis 310

Precepts of Universal Medical Practice. By Joseph Frank, Son of John Peter Frank. The Second Volume of the Third Part, the First Section, containing the Treatise on Diseases of the Intestinal Canal. By Frederic Augustus Benjamin Puchelt, Privy Counsellor to the Grand Duke of Baden.

Art. IV.—Praktische Monographie der Bandwurmkrankheit durch zweihundert sechs Krankheitsfälle erläutert. Von Dr. Andreas Ignaz Wawruch. Mit einem Vorworte von Dr. Ignaz Rudolf Bischoff . . . 319

Practical Monograph on Tapeworm; illustrated by Two Hundred and Six Cases of the Disease. By Dr. A. I. Wawruch. With a Preface by Dr. J. R. Bischoff.

Art. V.—Guy's Hospital Reports. Second Series. Nos. I, II, and III. April 1843 to April 1844 . . . . . . 332

No. I. 1. Mr. Taylor on a Case of suspected Irritant Poisoning . . ib.

2. Dr. Lever's Observations on Pelvic Tumours obstructing Parturition . 333

3. Dr. Chevers' Inquiry into the Causes of Death after Injuries and Surgical Operations . . . . . . ib.

4. Dr. Chevers on the Structure, Functions, and Diseases of the Coronary Arteries of the Heart . . . . . 334

5. Mr. King on the Digestive Solution of the Œsophagus . . 335

6. Dr. Hughes on a Case of Glanders in the Human Subject . . ib.

7. Mr. Poland's Report of Cases of Hernia at Guy's Hospital, from Sept. 1841 to Dec. 1842 . . . . . . 336

PAGE

8. Drs. Bright and Barlow's Observations on Patients whose Urine was Albuminous . . . . . . 336
9. Dr. Rees's Observations on the Blood, with reference to its peculiar Condition in the Morbus Brightii . . . . ib.
10. Mr. Browne's Report of Cases of Fever . . . 337

No. II. 1. Dr. Addison's Observations on Pneumonia and its Consequences . ib.
2. Mr. B. B. Cooper's Observations on Lithotomy . . . 339
3. Dr. Williams on the Pathology of Cells . . . ib.
4. Mr. Williams on a successful Case of Extirpation of the Superior Maxillary Bone . . . . . . 340
5. Mr. King on a Feculent Discharge at the Umbilicus, from Communication with the Diverticulum Ilei . . . . . ib.
6. Mr. Key on the Extirpation of Ovarian Cysts . . . ib.
7. Mr. Rose and Dr. Oldham on a Case ef extra-uterine Fetation . ib.
8. Dr. Lever on Cases of Puerperal Convulsions . - . . ib.
9. Report of Cases of Stricture of the Urethra, Retention, and Extravasation 341

No. III. 1. Dr. Lever's Cases of Pelvic Inflammation, with Abscess, occurring after delivery . . . . . . ib.
2. Mr. Taylor on Cases of Poisoning . . . . 342
3. Dr. Hughes and Mr. Cock on Paracentesis Thoracis . . 343
4. Dr. Oldham on Polypus Uteri, and its Coexistence with Pregnancy . 344

Art. VI.—1. Om den spedalske Sygdom (Elephantiasis Græcorum). Af Berglaege C. W. Boeck . . . . . . 346
On the Elephantiasis Græcorum, or Spedalskhed of Norway, &c. By C. W. Boeck, Physician to the Mines at Kongsberg.

2. Iagttagelser om Spedalske i St. Jörgens Hospital i Bérgen i 1841. Ved Corpslæge D. C. Danielsen i Bergen . . . . ib.
Observations on the Patients affected with Lepra in St. George's Hospital in Bergen. By Dr. Danielsen.

Art. VII.—1. On Ankylosis or Stiff-joint; a Practical Treatise on the Contractions and Deformities resulting from Diseases of the Joints. By W. J. Little, m.d. . . . . . . 353

2. Traitê Pratique de Pied-bot, de la fausse Ankylose du Genou et du Torticolis. Par V. Duval . . . . . . ib.
A Practical Treatise on Club-foot, False Ankylosis of the Knee, and Wry-neck. By V. Duval.

3. Du Traitement des fausse Ankyloses et de la Contracture des Membres par la Compression, aidée de l'Extension, sans l'Emploi de la Tenotomie, avec quelques Reflexions sur ce dernier mode opératoire. Par M. Dancel . ib.
On the Treatment of False Ankylosis and Contraction of the Limbs by Compression aided with Extension, without the Performance of Tenotomy, with some Reflections on the latter operation. By M. Dancel.

Art. VIII.—Medico-Chirurgical Transactions. Vol. XXVI . . 364

Art. IX.—1. Om Formen af Nordboernes Cranier. Af A. Retzius. Mit eenige bekorting vertaald door J. van der Hoeven, m.d. Tijdschrift voor Natuurlijke Geschiedeniss en Physiologie, Tiende Deel . . . 372
On the Form of the Skull in the Natives of the North [of Europe]. By A. Retzius. Translated [from Swedish into Dutch], with some Abbreviation, by J. van der Hoeven, m.d.

2. Uber die Ureinwohner von Peru. Von Dr. J. J. von Tschudi . . ib.
On the Original Inhabitants of Peru. By Dr. J. J. von Tschudi.

3. Bijdragen tot de Natuurlijke Geschiendeniss van den Negerstam. Door J. van der Hoeven, Med. Dr. Gewoon Hoogleeraar aan de Leidsche Hoogeschool enz . . . . . . . ib.
Contributions to the Natural History of the Negro-race. By J. van der Hoeven, m.d., Ordinary Professor in the University of Leyden.

Art. X.—Die Krankheiten der Bauchspeicheldrüse, etc. Von D. H. Claessen 388

The Diseases of the Pancreas. By Dr. H. Claessen.

PAGE

ART. XI.—A Practical Treatise on Diseases of the Eye. By WM. JEAFFRESON, late Surgeon to the Bombay Eye Infirmary, in the Hon. East India Company's Service . . . . . . 405

ART. XII.—Traité complet de l'Anatomie, de la Physiologie, et de la Pathologie du Système Nerveux Cerebro-spinal. Par M. FOVILLE. 1re Part.—Anatomie . 423
A Treatise on the Anatomy, Physiology, and Pathology of the Centre of the Cerebro-spinal Nervous System. By M. FOVILLE. First Part—Anatomy.

ART. XIII.—The Anatomy and Philosophy of Expression, as connected with the Fine Arts. By Sir CHARLES BELL, K.H. . . . . 442

ART. XIV.—1. Histoire d'un Opération d'Anus Artificiel pratiqueé avec succès par un nouveau Procédé, dans un cas d'absence congéniale de l'Anus; suivie de quelques Réflexions sur les Obturations du Rectum. Par J. Z. AMUSSAT. 452
History of an Operation for Artificial Anus, successfully performed by a New Method in a case of congenital absence of the Rectum. By J. Z. AMUSSAT.

2. Mémoire sur la Possibilité d'établir un Anus Artificiel dans la Région lombaire sans pénétrer dans la Péritoine. Par J. Z. AMUSSAT . . ib.
Memoir on the Possibility of establishing an Artificial Anus in the Lumbar Region without opening the Peritoneum. By J. Z. AMUSSAT.

3. Deuxième Mémoire sur la Possibilité d'établir un Anus Artificial dans les Régions lombaires sans ouvrir le Péritoine. Par J. Z. AMUSSAT. . ib.
Second Memoir on the Possibility of establishing an Artificial Anus in the Lumbar Regions without opening the Peritoneum. By J. Z. AMUSSAT.

4. Troisième Mémoire sur la Possibilité d'établir une Overture Artificielle sur le Colon lombaire gauche sans ouvrir le Péritoine, chez les Enfants imperforés. Par M. AMUSSAT . . . . . . ib.
Third Memoir on the Possibility of establishing an Artificial Opening in the left lumbar Colon without opening the Peritoneum in Infants born with Imperforate Anus. By M. AMUSSAT.

5. Du Cancer du Rectum et des Opérations qu'il peut réclamer, Parallèle des Méthodes de Litré et de Callisen pour l'Anus Artificiel. Par A. VIDAL (DE CASSIS) . . . . . . ib.
On Cancer of the Rectum and the Operations it may require; with a Parallel between the Methods of Litré and Callisen, for establishing an Artificial Anus. By A. VIDAL (DE CASSIS).

6. A Case of Carcinomatous Stricture of the Rectum, in which the descending Colon was opened in the Loin. By ALFRED JUKES . . ib.

ART. XV.—Life in the Sick-room. Essays by an Invalid . . 472

ART. XVI.—Recherches et Observations sur les Causes des Maladies Scrofuleuses. Par J. G. A. LUGOL, Médecin de l'Hôpital St. Louis . . 481
Researches and Observations on the Causes of Scrofulous diseases. By J. G. A. LUGOL, &c.

ART. XVII.—1. First Report of the Commissioners for inquiring into the State of large Towns and Populous Districts . . . . 492

2. Facts and Observations on the Sanitary State of Glasgow during the last year; with Statistical Tables of the late Epidemic, showing the connexion existing between Poverty, Disease, and Crime. By ROBERT PERRY, M.D. . ib.

3. The Glasgow Bills of Mortality for 1841 and 1842, drawn up by appointment and under the authority of the Lord Provost, Magistrates, and Town Council. By ALEXANDER WATT, LL.D., City Statist . . . . ib.

4. Observations on the Epidemic Fever of 1843 in Scotland, and its connexion with the destitute Condition of the Poor. By WILLIAM P. ALISON, M.D. . ib.

ART. XVIII.—Caloric; its Mechanical, Chemical, and Vital Agencies in the Phenomena of Nature. By SAMUEL METCALFE, M.D. . . . 513

ART. XIX.—On Dysmenorrhea and other Uterine Affections in connexion with Derangement of the Assimilating Functions. By EDWARD RIGBY, M.D. . 522

### PART SECOND.—Bibliographical Notices.

PAGE

ART. I.—Cases of Deformity from Burns successfully treated by Plastic Operations. By THOMAS D. MÜTTER, M.D. . . . . . 525

ART. II.—1. Researches on Phthisis, Anatomical, Pathological, and Therapeutical. By P. C. A. LOUIS, M.D. Second Edition, considerably enlarged. Translated by W. H. WALSHE, M.D. Printed for the Sydenham Society . 526

2. Thomæ Sydenham, M.D. Opera Omnia; Edidit G. A. GREENHILL, M.D. Impensis Societatis Sydenhaminæ . . . . . ib.

ART. III.—A Treatise on the Use of the Sympathetic Nerve and its Ganglions, with their Influence on various Diseases of the Anatomical and Pelvic Viscera. By T. B. PROCTER, M.D. M.R.C.S. . . . 528

ART. IV.—Ueber Wiedererzeügung der Knochen nach Resectionen beim Menschen. Von D. KAJETAN TEXTOR . . . . . . 529
On Regeneration of Bone in the human Subject after Excision. By D. KAJETAN TEXTOR.

ART. V.—Traité Analytique de la Digestion; considéré particulièrement dans l'Homme et dans les Animaux vertébrés. Par N. BLONDLOT, Docteur en Médecine, &c. . . . . . . . ib.
An Analytical Treatise on Digestion, considered especially in Man and in Vertebrated Animals. By N. BLONDLOT, Doctor of Medicine, &c.

ART. VI.—Human Physiology. By ROBLEY DUNGLISON . . . 530

ART. VII.—1. Chemistry, as exemplifying the Wisdom and Beneficence of God; being the Actonian Prize Essay. By GEORGE FOWNES, PH.D. &c. . 531
2. An Introduction to Practical Organic Chemistry; with references to the works of Davy, Brande, Liebig, &c. . . . . . ib.
3. The Chemical and Physiological Balance of Organic Nature: an Essay by MM. DUMAS and BOUSSINGAULT, Members of the Institute of France . ib.

ART. VIII.—The principal Offices of the Brain and other Centres. By JOSEPH SWAN . . . . . . . . 532

### PART THIRD.—Original Reports and Memoirs.

Report on the Progress of Toxicology, in relation to Medical Jurisprudence, Medical Police, Chemistry, and Pharmacy, for the years 1843-4. By ALFRED S. TAYLOR, Lecturer on Medical Jurisprudence and Chemistry in Guy's Hospital . . . . . . . 533

### PART FOURTH.—Medical Intelligence.

1. On Dislocations of the Astragalus. Reply to Mr. Turner's Reclamation published in the Provincial Medical Journal, Aug. 7, 1844 . . 565

2. Dr. CARPENTER's Reply to Mr. Wharton Jones, on the Functions of the White Corpuscles . . . . . . . . 569

BOOKS RECEIVED FOR REVIEW . . . . . . 284, 570

TITLE, INDEX, &c.

